# Alone in the wilderness—Cultural perspectives to the participants' motives and values from participating in a danish reality TV-show

**DOI:** 10.3389/fspor.2023.872485

**Published:** 2023-03-20

**Authors:** Søren Andkjær, Astrid Ishøi

**Affiliations:** Department of Sport Sciences and Clinical Biomechanics, Research Unit Active Living, University of Southern Denmark, Odense, Denmark

**Keywords:** outdoors, solo experience, friluftsliv, adventure, extreme sports, reality TV-show, media, cultural analysis

## Abstract

This paper focus on the participants in the Danish version of the reality TV-show Alone, named Alone in the wilderness (AIW), and seeks to explore *What motives and values are important to the participants in the TV-show (AIW) and how can the show be understood as a cultural phenomenon?* The study is qualitative with a design based on a triangulation of different methods: single interviews, transcripts of programs and autoethnographic notes. The analysis is inspired by a hermeneutic approach applying a 6-phased thematic analysis. The participants motives and values from their participation in the TV-show reflect ideas that may be related to the solo experience. On one hand the participants are motivated by the challenges of being alone in the wilderness and they value the possibility of personal development. On the other hand, they value nature and simple life in the outdoors, an experience that seems to grow more important to the participants as time goes. AIW is a competition and some of the participants are highly competitive aiming at winning the show, which however becomes less important during their stay in the wilderness. AIW as a cultural phenomenon reflects ideas and values related to an understanding of adventure and the Nordic tradition of *friluftsliv* (simple life in the outdoors) and can be related to theories on late modernity focusing on reflectivity and self-identity. The study presents new empirically based knowledge on the motives, values and experiences of people participating in AIW and it presents new theoretically based knowledge on how these motives, values and experiences can be understood as part of outdoor education and recreation and as a cultural phenomenon in late modern society.

## 1. Introduction

The American reality TV-show concept Alone is well known in many western countries and has become extremely popular in recent years. Denmark is no exception. Since 2016 five seasons of a Danish version of the TV-show, Alone in the wilderness (AIW), which is taking place in the northern Norway, has been shown on the national channel 1 (DR1) on Saturday evenings at primetime.

Reality TV as a genre has a long history (1) but the American TV-show Alone is a rather new concept, building on a series of reality shows that take place in natural landscapes. In the concept Alone a group of carefully casted, but otherwise “ordinary” people, are placed alone in the wilderness. Their challenge is to manage and survive using a limited amount of survival equipment. Except for medical check-ins, the participants are isolated from each other and all other humans. They must deal with inclement weather, hunger, and their own solitude, with the aim of staying out longer than their competitors, which they know nothing about while staying in the wilderness. They have an emergency phone to call for help and a “tap out” button to push if they want to be brought back to civilization.

The participants use video-record to self-document their experiences in solitude. From the large amount of material gathered the production company chooses the most interesting parts and creates a series of audience friendly episodes. The participants are carefully casted to ensure dynamic, excitement and emotions in each episode, and they are shown in different, often critical, and emotional situations. The episodes often contrast the participants' approaches to survival and each episode usually ends with one of the participants deciding to tap out and return home (2).

The participants are isolated from their friends and families, they are being exposed to extreme conditions in the wilderness, and they are being exposed to a large audience, depending on the producer team to present them in a reasonable positive way. In the Danish TV-show AIW there is no big price, and the winner primarily gets the experience of participating and the fame of winning.

Being alone in nature has fascinated people for many years and in many ways, and there seems to be an attraction and power related to the solo experience (3). In the TV-show AIW the solo experience becomes a mainstream phenomenon, produced by commercial production teams, and being globally exposed *via* modern mass media. Solo experiences thus are brought into people's living-rooms in the form of an international concept of reality TV-shows, and the participants become celebrities. It can be mentioned that in February 2020 the Danish TV-show AIW was the 7. most viewed TV program in Denmark (https://mir.dk/2020/03/10/alle-snakker-seertal/) and in 2019 the finale of AIW was seen by 862.000 viewers putting it on top 3 of the most viewed TV programs in Denmark at that time (https://www.dr.dk/om-dr/nyheder/ugens-tv-top-10-vi-var-vilde-med-vildmarken). The American reality concept has been exported to many different countries and millions of people watch the TV-show, following the participants and discussing how they cope with being alone in the wilderness and who will win? The notion of the untouched natural landscape in combination with the extreme situation—being alone in the wilderness—together with the element of competition seems to hold a great power of fascination. Like other reality TV-shows, it is good entertainment as it offers the possibility of personal identification with real characters (4).

The participants in AIW voluntarily choose to participate in the production of a reality TV-show that for a period radically changes their lives. With a focus on the participants in AIW we find it interesting to explore why people want to participate in the TV-show—exposing themselves to extreme challenges and to a vast and broad audience. In this paper we aim to question and understand: *What motives and values are important to the participants in the TV-show (AIW) and how can the show be understood as a cultural phenomenon?* These questions are interesting, as the TV-show, like other cultural phenomena, tells us about society and culture (5). Taking a closer look at the program and getting a deeper understanding of AIW might point to and perhaps challenge taken-for-granted understandings in our own culture. Challenging and gaining greater knowledge of these understandings may be seen as the foundation or first step towards change, e.g., according to the way people understand and use media and nature.

First, we introduce the background pointing to different traditions, trends, and cultures in outdoor education and recreation with a reference to theories on late modernity. After having lined up the materials and methods used in the study results are presented followed by a discussion with reference to different traditions, trends, and cultures in outdoor education and recreation, and drawing on Giddens theories on late modernity. The paper is rounded off with a conclusion and reflections on possible implications.

## 2. Background

In this section the ambition is to present the background for studying AIW as a cultural phenomenon. AIW is basically about being alone in nature and thus the starting point is literature on solo experiences. After that we present different traditions, trends, and cultures in outdoor education and recreation with a specific focus on adventure and friluftsliv, as two prominent categories relevant to understand motives and values in AIW. Theories on late modernity are presented to understand and explain AIW as a cultural phenomenon focusing on the diversity and development in outdoor education and recreation as part of modern society.

### 2.1. The solo experience

AIW basically is about being alone in nature, and as a cultural phenomenon it can be related to the solo experience. In literature we find numerous examples of the fascination and power related to being alone in the wilderness, often referred to as a solo experience. One good example is Henry David Thoureau, living alone for two years in the woods near Walden Pond in Concord, Massachusetts, who presented philosophical reflections on the relationship between “man” and nature and argued for the value of a simple life in nature (6). The Norwegian polar explorer Fridtjof Nansen, as an early ambassador for the Nordic concept of *friluftsliv,* explicitly advocated for the value of being alone in nature as an indispensable element in young people's character building including a critique of modern city life (7). Bjørn Tordsson, a contemporary Nordic pedagogic philosopher, described the core of friluftsliv and simple life in the outdoors as valuable existential experiences which—due to *nature's open indictment*—creates an opportunity to make meaning for the individual (8). Knapp and Smith (3) investigated the background to solo trips pointing to indigenous people, monks, and hermits, and to solo trips as rites of passage facilitating a special relation to animals and the natural world often involving spiritual experiences.

Solo experiences are used in therapeutic contexts with different groups, for example by women with the aim of personal growth (9), or in educational contexts as a pedagogical method to build up young people's self-awareness (10, 11). Quite a few studies point to the importance of participants volunteering to do a solo, the pre-solo mindset and to the facilitation process and the role of the instructor (10–13).

Personal development and the relation to nature seems to be closely related to the solo experience and AIW may be seen as a modern TV-show that builds on the history and tradition of the solo experience but staged as a reality TV-show. The TV-show follows the characteristics of reality shows (4), e.g., with a particular challenge to the participants as to how they perform and manage different roles (2). Following the overall research question in this paper, we will not include the aspects of media, performance, or the participants different roles.

### 2.2. Different traditions, trends, and cultures

Aiming at understanding motives, values, and the fascination of the reality TV-show AIW it seems relevant to investigate and further discuss different traditions, trends, and cultures in outdoor education and recreation, e.g., adventure and friluftsliv.

The concept of Adventure is generally understood as challenging situations in natural landscapes including risk, uncertainty, real consequences, and a demand for an active personal effort from the participants (14–18). Challenge is used pedagogically in an educational or therapeutic context with an expectation to achieve personal development leading to for example, increased self-efficacy, self-awareness, or resilience. The notion, although criticized (19–21), is that being exposed to challenge, one will learn about physical and mental limits and capacities and that this inevitably will lead to personal development.

Another prominent tradition is found in the Nordic tradition of friluftsliv (19, 22–24). Here the focus is on simple living, identification with nature and reflections on human-nature relations. The values of simple life in nature according to the Nordic tradition of friluftsliv are basic life in preferably unspoiled nature, plentiful of time, managing life with simple means. In a pedagogic context, friluftsliv and simple life in nature is often connected to democratic values, deep reflections, environmental awareness, and a close relation to tradition and a special place (19, 24, 25).

Adventure is overall understood as a global concept within outdoor education and recreation with roots in USA, GB, Australia, and New Zealand. Friluftsliv on the other hand is understood as a Nordic concept which relates to a special history and special values, but today seems to be spreading beyond the Nordic countries. The tradition of adventure, however, is not a straightforward and well-defined concept with studies pointing to different countries having distinct adventure cultures (18). The same can be said about the Nordic tradition of friluftsliv (19, 22, 23) pointing to the diversity and complexity within Outdoor Education and Recreation with more trends and values.

### 2.3. AIW as a cultural phenomenon

The TV-show Alone is understood as a cultural phenomenon that might tell us about human motives, habits, patterns of behavior and values related to nature and society. The TV-show is global and reflects different trends, which makes it an interesting and complex, new cultural phenomenon. Despite its global distribution and immense popularity, very little has been investigated in terms of its meaning to people and the relation to nature and to society.

Within Outdoor Education and Recreation, more concepts and cultural trends can be identified which seem to reflect geographical and cultural diversity as well as historical development. Culture in this context is understood as habits, patterns of behavior as well as values and motives for being active in natural landscapes. Within the last 20 years some studies have been published on the cultural aspects of Outdoor Education and Recreation (18, 19, 26–29). On one hand they point to the significance of Outdoor Education and Recreation to society and the ways in which traditions and programs reflect general values and trends in society. On the other hand, the studies point to social and cultural diversity within Outdoor Education and Recreation with more traditions, trends and cultures being promoted at different times and different places.

Theories on late modernity (30, 31) have been used to understand and explain the development and diversity within Outdoor Education and Recreation in modern society. Giddens and other social theorists' points to modern society being a continuation of modern institutional transitions and cultural developments. Giddens argue that the modernity of contemporary society is a developed, radicalized, “late” modernity which tend to be self-referring, instead of being defined in opposition to traditionalism, as in classical modernity. Modern societies are detraditionalized leading to enhanced reflexivity, both at the level of individuals and at the level of institutions. In the post-traditional order self-identity is reflexive and people are increasingly free to choose what they want to do and who they want to be leading to an increased focus on lifestyles. People thus need to create, maintain and revise a set of biographical narratives, social roles and lifestyles which can be seen in different aspects of modern life e.g., in their choices of outdoor activities and maybe also in their choice to participate in reality TV-shows.

Theories on late modernity, e.g., Giddens perspectives and focus on reflexivity and self-identity (30, 31), are often used to understand and explain motives and values in adventure and extreme sports (18, 19, 27, 32, 33) in a sociological and cultural perspective.

In this paper the focus is on the Danish version of the TV-show Alone in the wilderness (AIW), questioning the participants experiences in relation to their participation in AIW trying to understand their motives and values and why they chose to participate in the production of a reality TV-show that for a period radically changes their lives. By doing this we intend to get a deeper understanding of the inherent values and cultural meanings of AIW as a modern cultural phenomenon.

## 3. Materials and methods

This qualitative study intends to examine, identify and discuss motives and experiences among a particular group of people, and can be seen as an explorative case study (34–36) intending to produce new knowledge about a known phenomenon. The design aims to facilitate triangulation involving three different methods and sources of empirical material from AIW: (1) Qualitative single interviews (37) with two participants from season 3; (2) Transcripts of programs from season 1 and 2; and (3) Autoethnographic notes (38–40) from a participant from season 2 (co-author).

The empirical material (see Table 1) covers season 1–3, which are, if not identical, comparable in terms of structure, episodes, participants, and location. The two respondents (R1 & R2) in the single interviews, the transcript programs (P1–P7) and the auto-ethnographer (AI) were selected to have a broad material representing different seasons and different participants in terms of gender, age, and experience with outdoor activities. The empirical data can be criticized for containing different amounts of data from the different seasons and for a bias in relation to gender distribution (only men in transcripts). The total amount of empirical material, however, reflects a reasonable breadth in relation to both gender and the different seasons of the TV-show.

**Table 1:** Overview of the empirical material.

Form	Qualitative interviews		Transcripts		Autoethnographic notes	
Season	Season 3		Season 1 and 2		Season 2	
Year	2019		2017–2018		2018	
Info of the partici-pants	Name	Gender	Name	Gender	Name	Gender
Respondent (R1)	Female	Participant (P1)	Male	Auto-ethnographer (AI)	Female
		Participant (P2)	Male		
		Participant (P3)	Male		
Respondent (R2)	Male	Participant (P4)	Male		
		Participant (P5)	Male		
		Participant (P6)	Male		
		Participant (P7)	Male		

The two interviews ranged from 60 to 80 min in length and were audio recorded to ensure the accurate representation of participants' responses. The semi-structured interviews encouraged the development and elucidation of responses to understand participants' motives and experiences (41). The interviews were transcribed verbatim by the researchers before analysis.

Transcripts from the programs focus on the spoken language and not on nonverbal actions or appearances, as the purpose was to understand motives and experiences among the participants. Analytically it is important to recognize that the programs represent an edited version of an interpreted reality. First, the participants chose what to record, then the production-company selects which recordings to include in the TV-show, and finally, the material is being subject to interpretations.

The autoethnographic notes are made by the co-author (AI). The notes were written down shortly after the participation in AIW, to remember the experience but was not intended to be involved in scientific studies, which increases the credibility of the material (42).

The triangulation and the three different methods and sources of empirical material involved are chosen as it allows for a broad and thorough insights into the participants’ motives and experiences in relation to their participation in AIW. Triangulation involving different methods and empirical material was also chosen to avoid possible adverse implications of a researcher analyzing own autoethnographic data.

The analysis is informed and inspired by a hermeneutic approach (43, 44) aiming to interpretate and form a deeper understanding of the participants motives, experiences, and values focusing on both the individual parts and the entire material. The hermeneutic approach also includes a special emphasis on throughout the study to critically question own conjectures and preconceptions. This in combination with the triangulation of methods seems highly relevant according to the research questions to ensure the study`s validity and reliability.

To follow a relevant structure and process in the analytical work and to facilitate the hermeneutic interpretation the empirical material is interrogated using the 6-phased thematic analysis (45, 46): (1) familiarizing with data; (2) generating initial codes; (3) searching for themes; (4) reviewing themes; (5) defining and naming themes; (6) producing the paper. The analysis focuses on and reflects the research questions, while being open to the possibility of unforeseen themes emerging.

The empirical material was inductively coded by the researchers (47) with the two qualitive interviews initially coded and given priority, as they were expected to provide the most comprehensive empirical material with the greatest depth. In the next step the two other sources of empirical material were brought into the analysis to qualify the findings. In the analyzing process we aimed at identifying themes but also trying to assess the importance of the different themes and the participants' priorities in relation to the themes. Coding and themes identified were discussed in several rounds and verified in a final discussion by the two researchers with reference to the entire empirical material and with a critical perspective to own presumptions and conjectures. In addition to this the findings were peer reviewed by presenting and discussing them with two international colleagues (35). The accuracy of quotes and interpretations of participants' responses were verified by sending each respondent (R1 & R2) a draft copy of the paper for their review and approval (48).

## 4. Ethics

The transcripts of the selected seasons from the TV-show provides no ethical considerations. Regarding the qualitative interviews, these are anonymized, and the researchers have informed consent from the respondents.

The study and its data-management procedures were ethically approved by Legal Services, SDU, RIO (approval number 11.413). All data is managed in accordance with the GDPR regulations and are stored on a secure server at University of Southern Denmark.

The researchers represent no conflicts of interest in relation to the study.

## 5. Results

The analysis points to a variety of motives and important experiences linked to the participants’ participation in the TV-show AIW, which overall seem to reflect ideas and values related to the solo experience. The participants are generally motivated by the challenges of being alone in the wilderness and they value the possibility of personal development as well as the close relation to nature. Through the thematic analysis three rather broad and recurring themes emerged from the data: (1) challenge and personal development; (2) nature and simple life in the outdoors; and (3) competition and winning. The themes reflect the participants’ overall motives for participation and their important experiences from the time spent alone in the wilderness.

### 5.1. Challenge and personal development

The first theme, “challenge and personal development”, seems to be the most important motive for the participants. Challenges both physically and mentally are generally understood as positive and as a very important part of the experience. Facing challenges can be linked to the participants’ wish to test their capacities and thus meet expectations from themselves and others. The general understanding among the participants is that meeting challenges lead to personal development. A better understanding of oneself and one's own mental and physical capacity and boundaries is seen as the core of personal development. The participants generally recognize their participation as an individual and, to some extent, a selfish act.

The participation in AIW represents a series of very concrete physical and mental challenges to the participants. The physical challenges include having the skills to manage the situation, such as make fire, build a shelter, and provide food to survive in the wilderness. Perhaps even more important is the mental challenge of being alone, having to deal with solitude and one's own thoughts, missing friends and family and coping with hunger and fear in an uncertain and extreme wilderness situation. The uncertainty combined with the real consequences of failing is an important part of the experience of challenges:

*“Out here, everything becomes a little more extreme, because the consequences of succeeding and failing are a lot greater than usual.”* (P1).

Most of the participants see their participation as a kind of test, where they get the opportunity to seek and find their own physical and mental boundaries. The understanding is that facing challenges can tell them if their capabilities, skills, and mental strength are in line with their own self-understanding and self-image:

*“It was also a kind of study into myself and my own skills. A study into what you contain as a human being, what one really can and cannot do, but especially what you can do that you don`t think, you can do. So, the thing about testing boundaries was also a rather important factor in wanting to participate.”* (R2).

The participants generally understand themselves as rather skilled outdoor persons and have high expectations to their own capabilities. Quite a few of them emphasize that their expectations to themselves and their understandings of their own competencies are important parts of their motives for participation:

*“The most important thing for me is to get out there and see, if I can still do all the things, I once could.”* (P2).

*“But in general, being allowed to challenge myself to that extent, because I was naturally born with the ability to be very structured and systematic, so I spend a heck of a lot of time planning things when I need something and am therefore rarely pressured.”* (R1).

The experience of being physically and mentally challenged is closely linked to a notion of personal development, and the participants obviously have an expectation that experiencing challenges will lead to personal development. This is a very crucial element in their motives for participation and an important experience after the TV-show mentioned by most of the participants:

*“… the challenge I want to overcome the most and become good at is to be familiar with my own mind. For me, this is what it is all about—it is the fight against myself.”* (P3).

Personal development is understood as a process, where the challenging situations help the participants experience and push their own physical and mental boundaries, which help them to get a better understanding of themselves:

*“I have signed up, because I like the challenge of being myself and the challenge of being ‘man vs. wild’, right? (…) that you try yourself out, you test your limits: what can I really do? And you push those limits and it's an enormously positive experience, that you think, I can only do this and then you go a little further…”* (P3).

The personal development is closely linked to the participants’ reflections on themselves, on their skills and physical and mental capabilities, their relations to others and their life in general. The personal development, however, seems predominantly related to the mental perspectives of the challenges, meaning understanding, expanding, or accepting one's own mental capacities and limits. For some of the participants these reflections are very positive and important:

*“I don’t think that I as a person [am] a different human being than before my participation, but I am a much more conscious human being. Aware of my strengths and weaknesses and what I want with them and how I want to use myself as a tool, so in that way it has definitely changed me…”* (R1).

The participants generally are aware that their participation in AIW is a rather individualistic project. Their motives for participation are centered around themselves, and they seem to be open and reflective about this and to accept the selfishness of the project as well as the possible consequences for their families:

*“There is one dominant part of it, it is a personal challenge being allowed to do such an extreme and wild thing and a little bit selfish, to go out all alone and then have to manage yourself, and then the rest of the world and Denmark and your children too (…) they have to fend for themselves.”* (R1).

*“It is very much a dream come true. That I can run around and play Rambo for myself out there in nature, but… it is an enormously selfish act to leave one's family in order to realize myself. It's incredibly selfish.” (P5)*

The participants, however, also value nature and simple life in the outdoors, and this experience seems to grow more important to the participants as time goes.

### 5.2. Nature and simple life in the outdoors

Being in nature and living a simple life in the outdoors is important to the participants, as it affects them emotionally, mentally and makes them experience peace. Some of the participants are, due to the time in solitude in the wilderness, inspired to more existential reflections on their childhood and their daily life.

The participants enjoy a simple life in the outdoors and the basic and simple tasks necessary to live and thrive, such as making fire and building a shelter:

*“It is important to have peace in nature and in your soul when you are out there, in order to be able to plan and execute your things in the best possible way, so that you get the shelter, so that you get the food you need and so that you enjoy being there because that’s what it’s really about.”* (P4).

*“I really wanted to test my own limits and find out what it would be like to be alone for so long. (…) As well as trying to create a life and an everyday life in the middle of nowhere all alone. (…) And then I think it was very interesting that I had to manage by myself living in and by nature.” (AI)*.

The participants value the experiences of just being in nature, and nature and landscape seems to affect their emotions in different ways. Primarily nature and landscape help them experience humility and respect, but it also affects them mentally and makes them experience joy and peace. They experience nature and landscape as both beautiful and very powerful, leaving them with a feeling of being a small and fragile part of nature:

*“Nature and the overwhelming landscape, certainly help creating this atmosphere. This huge lake, the huge mountains, and those vast expanses. You just sit there and acknowledge that you are tiny, and you are here at the mercy of nature, and you only get what nature thinks you deserve, in some way. There is no giving at the doors and there are no shortcuts.”* (R2).

Being in nature for a long time affects their level of stress and their mental health, and some of the participants mention that they use nature this way in their daily life:

*“Nature can offer something that (…) indoors cannot. It gives another kind of peace of mind for me, (…) a better foundation for reflecting (…) and being able to sit and look out at the weather, on the water, in the fire, you can suddenly do that for many hours and then still think you are doing something.”* (R1).

Some of the participants, while being alone in the wilderness, seem to experience a particular connection to the place, which makes them think of their childhood where they had a special relation to nature. These experiences lead to more existentialistic reflections pointing to a more existentialist cohesion with nature and landscape:

*“For me, nature means a place I belong to.”* (P5),

*“Nature has been my sanctuary since I was young. I started running out into nature, when I needed to be alone, and it has given me a sense of security in nature…”* (P1).

The experiences alone in the wilderness make the participants reflect on simple life in nature as a contrast to their daily life both according to social relations, time, and materiality. Basic life alone in nature with plenty of time and only few material things seem to bring forth rather deep reflections on their daily life and life values. The participants appreciate the simple life and emphasize the time open for reflections, the calmness and the value of basic outdoor activities giving new perspectives to their daily life and their life values:

*“I think it evokes some thoughts in us about true values, and it’s a bit back to basic, it’s like, away with the phone, now let’s see each other, and you can live simpler, you can appreciate some things by being primitive. We do not need to have two boats or two cars or a holiday home in each part of the country.”* (R2).

*So, I didn't for a second miss all the communication options we have today. Purely materialistically, I felt I had everything I needed!” (AI)*.

The reflections on a simpler life with less focus on materialism are positive and valuable and some of the participants mention that they want these experiences and reflections to continue after their stay in the wilderness. Due to their solo time, some of the participants want to make radical changes in their daily life, and the experiences and reflections give basis for both self-criticism and to a kind of criticism to modern society and culture:

*“And that situation taught me a lot of things, partly in relation to thinking carefully and also thinking ahead [about environmental matters].”* (R1).

*“I am fascinated by the faith that our ancestors had here in the north and the approach they had to living in and with nature. (…) This basic idea of taking things back to a simpler level, I sure can take that with me.”* (P6).

The reflections on simple life in the outdoors as contrast to daily modern life and the reflections on life values, seem to be most prominent as part of the participants’ reflections after they have returned home.

### 5.3. Competition and winning

Competition is a central part of the TV concept AIW, and the participant who manages to stay alone in the wilderness for the longest time wins the show. In AIW the winner does not win a lot of money, as in the original US-version of the TV-show where there is a price of half a million US dollars. Instead, the winner gains fame and recognition, which involves becoming a known person and maybe being able to exploit the fame commercially.

Most of the participants clearly express that the competition is important to them, and that they are well prepared, dedicated and that they believe they will win the TV-show:

*"I really think I can win this. I have gone into it with a very positive attitude, and I feel I have control of my gear and I have control of myself. And I would almost go so far as to say I win.”* (P7).

*"I am a competitive person. I wanted to … yes participate in this competition and I really wanted to win it, I really did.” (AI)*

Most of the participants mention the element of competition as a very important part of their motivation to apply for AIW, and they generally have high expectations to their performance and chances to win. It seems, however, that the element of competition becomes less important over time, while other values, such as challenge, nature, and simple life, come significantly more into focus:

*“I experienced that I got up every day and was really happy* (…) *And one of the best things was that I think almost from the second, I was alone, it just did not matter with the competition. I didn’t have the urge I had, the three days we were at bootcamp, to win, it just disappeared. I still wanted to stay there for a long time, but it was not to win, it was no longer the criterion for success. And … it was just insanely liberating … and well, it was just nice to feel like that.”* (AI).

*“So when I left, both the goal of winning and knowledge, learning and experience were important, but if I had to say what is most important, I think, I have to honestly admit, it was probably winning, which weighed the most, but quite quickly, I don't know how long it takes (…) then it starts to be the other parts that (…) start to weigh more.”* (R1).

## 6. Discussion

Results show three main themes important to the participants' motives and experiences from their participation in AIW: (1) Challenge and personal development, (2) Nature and simple life in the outdoors, and (3) Competition and winning. Challenge and personal development seem to be an overall important motive and represent valuable experiences to the participants. Nature and simple life in the outdoors is highlighted by most of the participants to be the most important experience especially after their participation. Competition and winning seems to be an important motive prior to the participation, but more participants experience a change over time towards a greater focus on nature and simple life in the outdoors. The themes can be related to the literature and understandings of solo experiences and seem to reflect different trends and values found in outdoor education and recreation. Especially adventure and the Nordic tradition of friluftsliv seem to be prominent features that can be identified in the TV-show.

In the discussion the three main themes from the results will be discussed in relation to solo experience, different traditions in outdoor education and recreation and to theories on late modernity.

### 6.1. AIW as a solo experience

The participants are alone in the wilderness for a longer period and participation in AIW obviously is a kind of solo experience. In this way it seems to reflect the fascination and power related to being alone in the wilderness as it is found in the literature on solo experiences. The participation is voluntary, and it includes an appreciation of the untouched and unspoiled landscape as a value of a simple life in nature (6, 7). It also seems obvious that participating in AIW in different ways is linked to an understanding that participation can lead to personal development (7). In literature the personal development is often connected with a notion of character building by coping with difficult physical or technical challenges and daring to cross personal and mental boundaries (14–18). In AIW, however, it seems that the personal development is predominantly associated with the mental challenge of being alone for a longer period.

There are obvious differences between the solo experience as it is presented in literature and the TV-show AIW. The biggest difference might be the entire setup and organization of AIW which is media driven and includes a production team and broadcast to an unknown number of viewers. The show obviously does not have an explicit educational or therapeutic purpose, but rather serves a commercial purpose that lies outside the participants. This means that the participants knowingly are in a situation where they on one hand are alone and on the other hand will become publicly available to a larger audience.

AIW can be understood as a new way of thinking and practicing the solo experience, which is not pedagogic or therapeutic motivated, but where technology and media use and appearance is essential (2). In this situation the participants may have other motives and values for participation which point in different directions in relation to well-known concepts or cultures of outdoor activities.

### 6.2. AIW and different traditions, trends, and cultures

The participants motives, values, and experiences from their participation in the TV-show and their solo experience point in different directions and can be related to different traditions, trends, and cultures in outdoor education and recreation.

A solo experience is challenging in many ways and challenge and personal development are crucial parts of the motives and experiences important to the participants in AIW. The participants in AIW are generally motivated by the risks, the uncertainty, and the challenges they need to face while being alone in the wilderness. They also have expectations that their participation might have an impact according to personal development and that it will affect their everyday life in a positive way. This points to adventure as a concept (14–18), and it can be argued that AIW reproduces central ideas and values from adventure focusing on risk, uncertainty, challenge, and personal development. The participants are highly motivated by these values, and they see their participation as an individualistic project which have certain costs for their family and others. The expectations to the effects or significance of the personal development can be discussed (19–21) not least in relation to the long-term effects and durability of possible changes. Within the framework of this study, it is not possible to say whether the participation has led to personal development and in any way has changed the lives of the participants.

The participants are, while being alone in the wilderness, living a rather simple life in the outdoors and they generally value nature, landscape, and the basic tasks. The simple life in nature, the relation to nature and the reflections on life values are important to the participants and seem to reflect values of simple life in nature pointing to the Nordic tradition of friluftsliv (19, 23, 24) as it is lived and passed on in the Nordic countries.

This appreciation of nature and the simple life in the outdoors may seem like a paradox with reference to the entire set-up and staging of the TV-show. It could thus be argued that, due to mass media, commercialization and the element of competition, the proponents of friluftsliv, philosophers such as Nansen, Næss, and Faarlund [see e.g., (7, 22, 49)], would not approve of AIW and they would most likely not see it as a reflection of friluftsliv and simple life in nature. In that perspective it may seem paradoxical purposefully and voluntarily to seek the value of simple life in nature by participating in an international reality TV-show—instead of just going out and live a simple life in nature without cameras, production-team, audience, and competition. The appreciation of nature and the simple life in the outdoors is understood as a central motive and value related to participation in AIW but in a cultural perspective this obviously presents a paradox.

The element of competition represents a perspective to AIW that in many ways seem different from the concepts of adventure and friluftsliv. Extreme sports are often described and defined by a number of characteristics, e.g., the wilderness setting, the extreme conditions, and the element of competition (33, 50, 51). Due to these characteristics, obviously being central parts of AIW, it is possible to understand AIW in the light of extreme sports reproducing central elements that in a sociological perspective define extreme sports: the unfamiliar environment, the concrete risks, the uncertainty, and in particular the competition (33, 50).

Extreme sports, e.g., whitewater kayaking, extreme skiing, or Base-jumping, however, are in literature often described and related to experiences of speed, action and more thrilling and sensational experiences and challenges (33, 50, 51). This is very different and far from the participants experiences in AIW where the atmosphere and energy predominantly are characterized by calmness, routines and by a different relationship to time.

Competition and the values related to extreme sports seems to become less important to the participants during their time in the wilderness and afterwards. The obvious attraction and possibility of winning the competition seems to fade and be replaced by other values related to nature, the process and just being in nature. A possible explanation might be that AIW does not involve all the central characteristics of extreme sports, and especially lacks the experiences of speed, action, thrill, and sensation and perhaps even more important that throughout the process other values stand out more clearly and become valuable.

Aiming at understanding the participants motives and values and especially the process of their stay in the wilderness and the changes that emerge an interesting reflection seems relevant. Before their participation many of the participants are highly attracted to and motivated by the competition and the possibility to win the show as well as the prospect of personal development that can affect their identity and future everyday life. However, as they are alone in the wilderness and after the show when they reflect on their participation, they seem to value the basic life and just being in nature. The difference and the development can be understood as a movement from motives predominantly related to a personal outcome towards motives and values predominantly related to the experience or process of just being in nature which can be related to friluftsliv and simple life in the outdoors (19, 23, 24).

### 6.3. AIW and late modernity

Giddens perspectives on late modernity have been used intending to understand and explain motives and values in adventure and extreme sports in a sociological and cultural perspective (18, 27, 32, 33). It thus seems relevant to discuss the TV-show AIW and the participant's values and experiences in relation to these theories.

Giddens characterized late modern societies by their dynamic and rapidly changing character as well as by an overall element of globalization (30, 31). The TV-show Alone, as an American concept, has been exported to many countries which points to globalization as a central element reflecting late modernity. The element of competition related to and understood in the light of extreme sports (33, 50, 51) can be seen as another example of the reproduction of global modern values, placing emphasis on individuality and progress.

The prominent element of challenge and personal development in AIW represents possibilities for the participants to be tested in extreme situations, and thus achieve an expected personal development. Important issues in AIW thus are the participants' process and their efforts and challenges to revise, create and maintain their own identity and lifestyle, which can be understood as a focus on reflexivity and self-identity (31). The participants in AIW experience that their participation is an individual project and responsibility which offers them a possible way through challenge and personal development to create self-identity. The participantś motives for participation focusing on adventure and challenge thus points to AIW as a cultural phenomenon reflecting features and values from late modernity.

Not all findings, however, can be understood or explained in the light of the theory of late modernity. Nature and simple life in the outdoors points in another direction and makes it a bit more complex. Simple life in nature, related to the Nordic concept of friluftsliv (8, 19, 22, 23) reflects ideas and values on nature, time, simplicity, social relations, and a relation to place and landscape that to some point contrast theories and understandings of late modernity. The values point to a more traditional society where the process, tradition, and craftsmanship are central cultural values. The participants obviously appreciate the basic values that contrast with their daily cultural and modern life. Simple life in nature including deep reflections on daily life and life values can thus be seen as a contrast, or maybe even an element of criticism to modern society. These reflections are not prominent parts of the participant's motives for participation rather a product of being alone in nature for a longer period. The participantś reflections on life values, however, can be linked to an understanding of self-identity and reflexivity leading to possible changes in lifestyle. It can be argued that these reflections are necessary in late modern society (30, 31).

AIW can be understood as a complex cultural phenomenon that reflects values and complexities from late modernity with a focus on globalization, reflexivity and self-identity often related to an understanding of adventure and extreme sports (18, 27, 32, 33). At the same time AIW presents radically different ideas and values, which point to a more traditional view on history, culture, and society. These ideas and values seem to represent a potential criticism to everyday life and modern society as it is found in the Nordic concept of friluftsliv (8, 19, 22, 23).

## 7. Conclusions and implications

The participants in AIW voluntarily chose to participate in the production of a reality TV-show that for a period radically changes their lives. We initially asked: *What motives and values are important to the participants in the TV-show (AIW) and how can the show be understood as a cultural phenomenon?*

Participating in the TV-show AIW can be understood as a modern version of a solo experience staged as a mediated reality TV-show. The thematic analysis highlights three themes: (1) challenge and personal development; (2) nature and simple life in the outdoors, and (3) competition and winning. Challenge and personal development seem to be the most important motive for the participants prior to their participation in AIW. Nature and simple life in the outdoors is important to many of the participants especially as a valuable experience after their participation. The competition and possible chance of winning is an important motive to many of the participants prior to their participation but seems to become less important as the participant`s lives in solitude unfold.

AIW can be understood as an extreme popular cultural medialized and global phenomenon that relates to different trends and traditions in society, and which produces and reflects central ideas and values in modern society today. AIW on one hand reflects and presents values related to an understanding of adventure focusing on challenge and self-development, and on the other hand reflects values on nature and simple life in the outdoors, pointing to the Nordic tradition of friluftsliv.

Overall AIW can be seen as a cultural phenomenon that points to and can be explained by theories of late modernity (30, 31). AIW reflects the radical changes in social life in modern society with a special perspective to globalization, reflexivity, and self-identity. At the same time values related to nature and simple life in the outdoors are being produced and reflected. These values seem to contrast theories and understandings of late modernity pointing to a more basic lifestyle, and they can be understood as a potential criticism to everyday life and society. The TV-show AIW thus can be understood as a complex modern medialized cultural phenomenon that points to values in late modernity but also holds motives and values that points to other ways of living and other lifestyles.

The study on AIW presents new knowledge about the participants and the TV-show, which serves more purposes. The study presents new empirically based knowledge on the different motives, values and experiences of people participating in a popular Danish reality TV-show. It presents new theoretically based knowledge on how these motives, values and experiences can be understood as part of outdoor education and recreation and as a cultural phenomenon in late modern society.

The study can be seen as a way to apply a critical cultural perspective to everyday cultural phenomena such as TV-shows and outdoor activities and thus serve as an example of how cultural analysis and sociological theories can be used to understand the deeper complexities of everyday phenomena, and how these may produce and represent different motives, ideas, and values. This kind of knowledge is often under-prioritized but may be highly relevant both in an educational, sociological, and public health context.

The three prominent themes point in different directions and seem to reproduce rather different motives and values. AIW, as an example of a global and medialized reality TV-show, attracts a big audience and may impact people's perceptions and attitudes towards nature. Reality TV-shows like AIW, by virtue of their power of fascination and identification, have an impact on people's understanding of e.g., media and nature, which are often based on taken-for-granted understandings and expectations. The study, however, does not give answers to how these ideas and values are transformed to an audience and how this may affect them.

The reality TV concept Alone is a rather new cultural phenomenon and despite its global distribution and immense popularity, very little has been investigated and documented in terms of its meaning or significance to people and society. Due to the power of media and the great popularity of the TV-show, it has a huge potential to influence others (1, 4). The reach and fascination of modern media and the element of identification point to the impact of the TV-show AIW to be strong promoting cultural values in society which calls for an increased research interest in modern cultural phenomena such as reality TV-shows with a focus on meaning and significance, and with the use of different designs and methods.

## Data Availability

The original contributions presented in the study are included in the article/Supplementary Material, further inquiries can be directed to the corresponding author.
